# Haiti National Program for the Elimination of Lymphatic Filariasis—A Model of Success in the Face of Adversity

**DOI:** 10.1371/journal.pntd.0002915

**Published:** 2014-07-17

**Authors:** Roland Oscar, Jean Frantz Lemoine, Abdel Nasser Direny, Luccene Desir, Valery E. Madsen Beau de Rochars, Mathieu J. P. Poirier, Ann Varghese, Ijeoma Obidegwu, Patrick J. Lammie, Thomas G. Streit, Marie Denise Milord

**Affiliations:** 1 Ministry of Public Health and Population, Port au Prince, Haiti; 2 IMA World Health, New Windsor, Maryland, United States of America; 3 Hopital Ste. Croix, Léogâne, Haiti; 4 University of Notre Dame, Notre Dame, Indiana, United States of America; 5 Centers for Disease Control and Prevention, Atlanta, Georgia, United States of America; 6 CBM, Greeneville, South Carolina, United States of America; Ministry of Health, Kenya

Lymphatic filariasis (LF) is a mosquito-borne parasitic infection that causes lymphedema, elephantiasis, and hydrocele. Haiti is one of only four countries left in the Americas where transmission of lymphatic filariasis still occurs. The National Program to Eliminate LF (NPELF) was started in Haiti in 2000, and by 2005 a population of 1.6 million people in 24 communes, including the majority of high-prevalence communes, was targeted at least once for mass drug administration (MDA). An interruption in external funding at the end of 2005 paralyzed the program, but with new donor support the NPELF was able to scale up to achieve full geographic coverage, reaching more than 8 million people in 2012. The LF program in Haiti has faced many challenges, including political crises, hurricanes, a devastating earthquake, and a deadly cholera outbreak in the earthquake's aftermath. Despite these challenges, the NPELF and partners have persisted, and now the program is integrated with soil-transmitted helminth (STH) control, is national in scope, and provides appropriate supportive care for persons suffering from LF morbidity. Haiti serves as a model for successful program implementation in countries affected by political and social challenges and natural disasters.

## Introduction

Lymphatic filariasis (LF) is a mosquito-borne parasitic infection that is best known for causing elephantiasis and hydrocele, conditions that prevent affected persons from living full and productive lives and may isolate them from family and community [1], [2]. Haiti is one of only four countries remaining in the Americas where transmission of LF still occurs [3]. Given that a clear relationship between poverty and the occurrence of LF has been established and that Haiti has the highest poverty rate and poorest health indicators of the countries in the Western Hemisphere, it is not surprising that Haiti also has the greatest burden of LF infection and disease [4], [5]. The *Culex quinquefasciatus* mosquitoes that transmit LF in Haiti are adapted to breed in conditions in which the environment has been degraded, a description which applies to much of Haiti's countryside, but increasingly so in urban and peri-urban locales as well as in the areas affected by the aftermath of the earthquake early in 2010.

Significant progress has been made in the effort to develop a national LF program in Haiti, thanks to the highly motivated staff of the Ministry of Public Health and the Population (MSPP) and partners and critical funding support from the Bill & Melinda Gates Foundation, the United States Agency for International Development (USAID), the Centers for Disease Control and Prevention (CDC), CBM, the Inter-American Development Bank (IDB), the Abbott Foundation, PepsiCo, the Frank Eck Family Foundation, and other private donors. This manuscript will review Haiti's remarkable success in overcoming a host of challenges in the ongoing effort to eliminate LF, support persons disabled by LF, and control neglected tropical diseases (NTDs).

## Lymphatic Filariasis in Haiti—Defining the Problem

Studies to characterize the epidemiology of LF in Haiti were carried out in the 1970s and 1980s by Dr. Christian Raccurt and colleagues [6], [7]. These surveys, conducted prior to the development of antigen detection tests, indicated that LF was concentrated in the coastal plains. With the advent of rapid antigen detection assays, MSPP and partners were able to undertake a more extensive national survey, thanks to critical funding support from the Bill & Melinda Gates Foundation. In each of Haiti's 133 communes or districts (at that time), 100–250 schoolchildren (ages 6–10) were tested for circulating filarial antigen (CFA) using a rapid immunochromatographic (ICT) test [8]. The results showed that approximately 90% of the communes were classified as in need of mass drug administration (MDA) following the guidelines developed by the World Health Organization (WHO) (1% infection prevalence threshold), representing an at risk population of nearly 8 million people [9]. Subsequent surveys identified autochthonous transmission in low-prevalence communes [10]. These results, demonstrating that LF is more widely distributed than earlier surveys had indicated, may reflect low-level or seasonal transmission that was previously unrecognized, the expansion of *Culex* and LF transmission into previously LF-free areas, population migration, or some combination of all of these.

## Approach of the National Program to Eliminate LF

**Figure 1:**
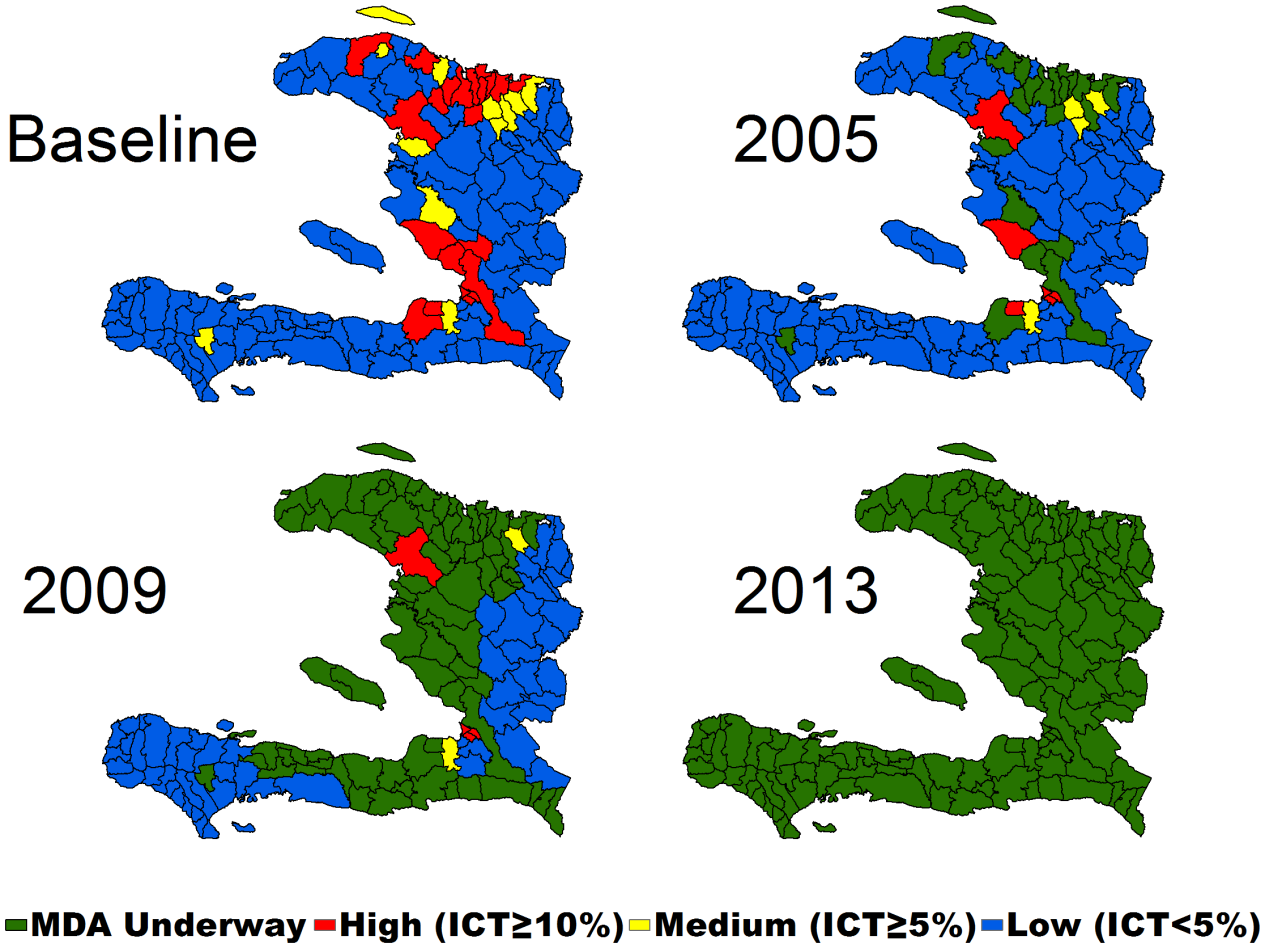
Mapping data based on initial antigen testing of schoolchildren and growth of the national program, Haiti, 2000–2013.

Following the completion of mapping, the MSPP established the National Program to Eliminate LF (NPELF) with three goals: (1) to interrupt transmission, (2) to reduce the suffering of persons with clinical and chronic manifestations of LF, and (3) to encourage positive health behaviors. During the first years of the NPELF, the main focus of the program was to interrupt transmission. To achieve this, communes were categorized according to their endemicity level as defined by the CFA surveys into high (≥10%), moderate (5%–9.9%), low (0.1–4.9%), and nonendemic communes (Figure 1). Treatment was targeted to the most highly endemic communes with a secondary goal of selecting communes in different geographic regions of the country. The initial plans called for doubling the population targeted for MDA each year for three years until full national coverage could be achieved, but because of financial and logistical limitations, this plan was modified after consideration of how to best maximize finite resources. Consequently, MDA activities were scaled up in the most highly endemic communes within the limitations imposed by the funding support available.

## Mass Drug Administration (MDA) and Scaling Up

MDA in Haiti first started in Léogâne under the umbrella of a CDC-funded demonstration project with diethylcarbamazine (DEC) and albendazole in 2000 [11], [12]. This project provided an opportunity to pilot social mobilization and drug distribution strategies for Haiti. Based on this experience, the NPELF, in partnership with the University of Notre Dame (South Bend, Indiana, United States), Hôpital Ste. Croix (Léogâne), CDC (Atlanta, Georgia, US), and Interchurch Medical Assistance (now called IMA World Health), expanded the MDA efforts to other regions of Haiti.

The approach used in most of the communes included a number of training sessions prior to each activity. Training for community leaders was conducted to sensitize and educate them about LF, including how to organize the MDA. Training sessions on health communication were organized in each community and focused on main messages related to LF treatment, transmission, and prevention, as well as the clinical manifestations of LF. Training was followed by an intense social mobilization campaign using available media including banners, posters, audio spots on local radio stations, and messages delivered by megaphones to inform and encourage the population to participate in the MDA [13]. Finally, just prior to the distribution, a team from the NPELF and local partners trained the distribution-post volunteers to carry out the MDA and to manage treatment-related adverse events [14]. Based on the Léogâne experience, it was decided to use distribution posts as the primary delivery model for MDA. Sites selected to serve as distribution posts included schools, markets, health facilities, churches, and local gathering places. Each post was staffed by three or four volunteers responsible for motivating the population to participate in MDA, registering participants, and distributing the drugs. Staff from the NPELF, partners, and local representatives of the MSPP provided supervision. Basic medications for treatment of minor adverse events (acetaminophen and ibuprofen) were provided at the distribution post, while, for more serious adverse events, persons were referred to health centers or the local hospital, where they were treated free of charge [14]. As per WHO guidelines, children aged less than two years and pregnant women were excluded from treatment. Reported coverage data by age and gender were collected from each distribution post, and MDA programs also were evaluated through knowledge, attitude, and practice (KAP) surveys, coverage surveys, and impact assessments [12], [15], [16]. In general, surveyed coverage exceeded 70% but was 10%–20% lower than reported coverage, at least in part because of differences between the population numbers derived from coverage surveys and the official population statistics that were used as the denominator.

During 2000–2002, only the commune of Léogâne was treated (census population: 157,000). In 2003, the NPELF and IMA World Health each targeted four new communes with a population of 492,000. In subsequent years, the partners added communes as shown in Figure 1 until, by 2005, a population of around 1.7 million people in 24 communes was targeted for MDA. Though this number only represented 19.6% of the total population at risk, the proportion of infected person targeted was much higher because of the initial decision to target the most highly endemic communes.

## Challenges to the Program

Haiti has faced many challenges that have affected the scaling up of the program. A political crisis enveloped Haiti beginning in 2003 that escalated into violence in 2004, with kidnappings and carjackings becoming commonplace in Port-au-Prince. In that period, one staff driver of the NPELF was shot and a principal administrator for the Hopital Ste. Croix program was killed by the random violence that seized Port-au-Prince, Gonaives, and other cities. However, even with the political unrest and violence spreading over the country in 2004 and 2005, the NPELF and partners continued MDAs. This persistence reflected the expressed desire of the treated communities and program staff to keep the program going. Despite the commitment of the NPELF, an interruption in external funding at the end of 2005 halted the program. Although the political situation was eventually stabilized by the introduction of a United Nations peacekeeping force, the social unrest and violence accelerated a longstanding pattern of outmigration among skilled health and other workers: the so-called “brain drain.” The impact of the interruption in funding on transmission was assessed in Léogâne; both microfilaremia and antigenemia prevalence increased, demonstrating that a single year of missed MDA can set the program back by up to two years in high-transmission settings like Léogâne [17].

## Reviving the NPELF

**Figure 2:**
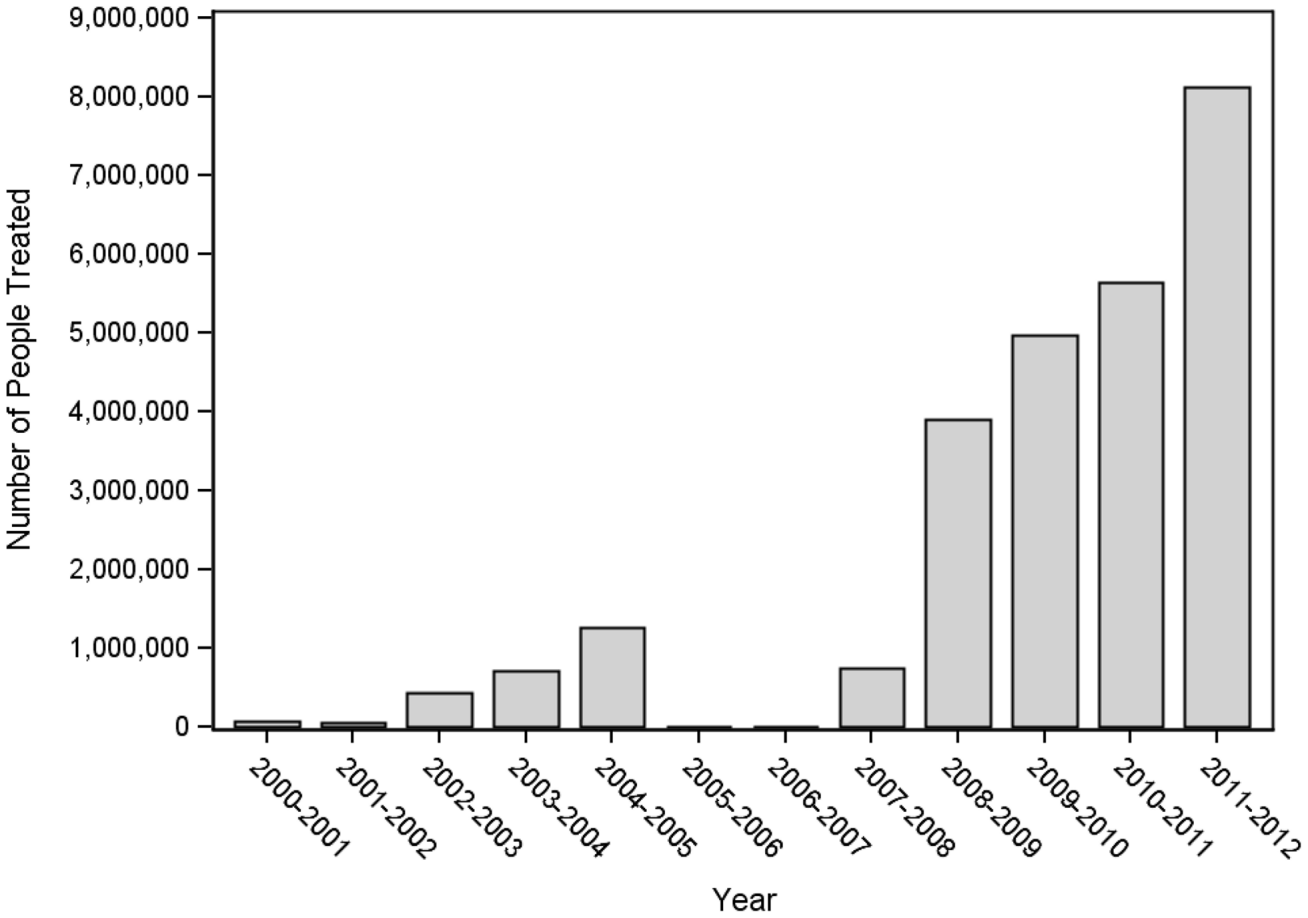
Number of people treated per year by program year.

Extensive advocacy efforts were needed to convince donors that the LF program in Haiti was providing health benefits and that it could succeed. With the funding stream restored in late 2006, the program was able to return to the level of geographic coverage attained in 2005. In addition, a new award from RTI/USAID also resulted from the increasing interest of the international donor community in the integration of delivery of preventive chemotherapy for NTDs. Both LF and intestinal helminth infections represent public health concerns across Haiti [8], [18]. The LF program delivers important public health benefits through the coadministration of albendazole with DEC [19]. The new funding allowed the NPELF to move away from a focus on communes with the highest endemicity toward a strategy focused on 100% geographic coverage across all departments (provinces). The NPELF reached 2.35 million people in 2008 and 4.5 million in 2009 (Figure 2). The growth of the program was accompanied by important economies of scale; the cost of annual treatment per person decreased to less than US$0.50, a dramatic reduction from the US$2.23 reported in the first days of MDA in Léogâne [11], [20].

With a more stable funding base, the NPELF developed plans to cover the entire country with MDA, with the exceptions of the metropolitan area of Port-au-Prince, again due to resource constraints, and the island of La Tortue, where two rounds of MDA have apparently interrupted transmission (Hemme et al., unpublished data; manuscript in preparation). Even as the program scaled up, natural disasters continued to present challenges. Four hurricanes hit Haiti in rapid succession in 2008, leading to widespread flooding and delays in implementing MDA in Gonaives and elsewhere. The 2010 earthquake coincided with one of the regularly scheduled meetings of NTD partners in Haiti. A staff member for one of the partners died in the collapse of his home, and several others were trapped in the wreckage of the meeting venue. The earthquake caused massive destruction in Port-au-Prince and throughout the West Department, killing more than 300,000 people and displacing more than a million more. On the heels of the earthquake's devastation, a cholera outbreak started in the center of the country during October 2010 and spread nationwide within a little over a month [21]. In the face of the enormity of the setbacks centered on the urban population area that still required initial implementation of MDA, the Pan American Health Organization (PAHO), IDB, and the Global Network for Neglected Tropical Diseases (GNTD) hosted a meeting of partners and donors to regroup and establish new plans for the program. The partners affirmed their support for NTD control and elimination of LF, and the donor community responded. With new funding for MDA in Port-au-Prince from CDC and others, Haiti was finally poised for the first time to achieve full coverage of the entire country with MDA, with a population in excess of 10 million.

By February 2012, MDA was completed in the final six communes comprising the metropolitan area, bringing the total number of people treated nationwide to 8.1 million (Figures 1 and 2). Due to uncertainty of the denominator size of the population of the greater Port-au-Prince area after the 2010 earthquake and the resulting migration, an MDA coverage survey was conducted, which confirmed 71% (95% CI 69%–74%) of the metropolitan population had swallowed a tablet distributed by MDA [22]. With the program in Port-au-Prince achieving adequate population coverage, all the current 140 communes of Haiti had been covered by at least one year of MDA.

## Technical Challenges

**Figure 3:**
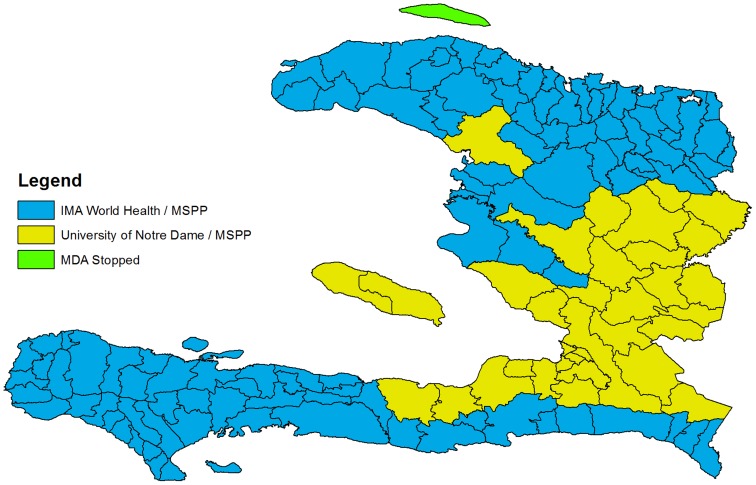
Delivery of MDA.

The realization of full national coverage fulfilled the first step toward successful elimination of LF from Haiti (Figure 3), but interrupting transmission of LF will require committed partners, a continued focus on fund raising, and, likely, innovative intervention strategies. As noted above, inconsistent funding and irregular MDAs represent a significant and ever present threat to the success of the program [17]. In addition, based on the results from Léogâne, where there is evidence of ongoing transmission despite more than five years of good coverage [12], interrupting transmission in the areas of highest prevalence will require either more than five years of MDA or new approaches to increase the effectiveness of MDA. In Léogâne, persons who consistently fail to participate in MDA have been suggested to represent a reservoir of infection [23], [24]. Working in India, Cantey and colleagues have shown that introduction of community-wide lymphedema treatment programs can increase MDA compliance [25]; perhaps this approach can be adapted to the program in Haiti. If compliance can be improved, MDA could be conducted twice per year to hasten the interruption of transmission [26].

In principle, DEC-fortified salt represents an alternative approach that can facilitate elimination of LF [27], [28]. In addition, when co-fortified with potassium iodate, its distribution assists in the elimination of iodine deficiency disorders as well. DEC-fortified salt also has the potential to be less expensive than MDA and, based on pilot studies in Haiti and elsewhere, more effective as well [28], [29]. In fact, achieving broad distribution of DEC-fortified salt will significantly impact the need for and duration of MDA programs, potentially achieving a material reduction in overall program costs. The local manufacture of co-fortified salt has been ongoing since 2005, with Hopital Ste. Croix, University of Notre Dame, and other partners assisting with processing of fortified salt at a MSPP-directed facility in Port-au-Prince. Distribution of 300–500 metric tons of salt per year (1%–2% of national consumption) is focused on Léogâne and nearby communities.

Recent developments within the salt project raise the prospect of a significant increase in the production and distribution of co-fortified salt, including the establishment of a three-year technical services agreement with Cargill Salt, an upgrade in the salt production facility to allow production of 3,000 metric tons per year, and development of a National Salt Strategy to regulate food-grade, fortified salt. All of these steps will facilitate a transition to processed food-grade salt, with the potential to greatly assist the sales and marketing of DEC-fortified salt.

Another possible way to accelerate the elimination of LF could be through the use of insecticide-impregnated materials distributed to control and eventually eliminate malaria from Hispaniola [30]. With support from the Global Fund, more than 3 million long-lasting insecticide-treated nets (LLIN) have now been distributed across Haiti. Although *C. quinquefasciatus*, the vector of LF, is thought to be less affected by LLIN than *Anopheles* spp., it is likely that such a program may, nonetheless, reduce human-mosquito vector contact and contribute to interruption of LF transmission. Other vector control methods, including source reduction and the use of polystyrene beads to interfere with larval development, have not been widely implemented in Haiti, primarily for cost and practicality considerations.

## Morbidity Program

Though targeting LF morbidity was originally described as the second pillar of the global program to eliminate LF, establishing strong systems for providing care for patients with morbidity due to LF has been a challenge for many countries [3]. The second goal of the program was addressed initially in Haiti by providing lymphedema management support at two referral centers, Hôpital Ste. Croix in Léogâne and Hôpital Sacre Cœur in Milot (near Cap Haitien). Since the beginning of the national program, more than 1,500 patients have received care for lymphedema, and more than 50 health workers were trained. Clinical care was supplemented through home visits and by establishing patient support groups [31]. These programs suffered from lack of continuity because of the funding problems noted above. One clinic was closed, and the NPELF was not able to extend services to other communities. New donor support has opened the door to the continuation of patient support groups with a focus on basic self-care through hygiene promotion and the provision of livelihood opportunities. Ten self-help groups of 20 people (primarily women) have been formed in Léogâne, and work is underway to establish patient groups in Carrefour. With a renewed focus on patient advocacy, these programs should reinforce the MDA program by increasing awareness of the causes of lymphedema and elephantiasis and reduce the social stigma faced by those who suffer from the disease.

Surgery using appropriate technique is necessary to provide a long-term solution for patients with hydrocele. A training program was established at Hôpital Ste. Croix to provide experience in the surgery technique pioneered by Professor Joaquim Noroes in Recife, Brazil, and recommended by WHO, in which the entire tunica vaginalis is excised [32]. To date, more than 2,000 hydrocele surgeries have been performed in Léogâne. Building off this experience base will require more training of local surgeons and donor support to reduce the cost of surgery for affected patients.

## Looking Forward

The MSPP in Haiti is determined to interrupt LF transmission and control intestinal helminths using MDA in combination with other approaches. Despite a host of challenges, the LF program stands as an exceptional public health success in Haiti. MDA is popular both with MSPP and communities because of the significant public health benefits that it delivers. Even in settings that are characterized by political and civil strife and natural disasters, MDA can continue to provide a platform for delivery of health services to impoverished communities. The appeal of this message extends beyond Haiti to other countries facing crushing poverty and civil strife. LF and other NTD programs represent an inviting opportunity to engage communities that frequently lack organized public health activities and to provide them with significant public health benefits. Haiti is prepared to meet the technical challenges that need to be addressed to end transmission of LF, to extend care to all those already affected by this disease, and to extend these successes to other public health problems facing the country.

## References

[1] Coreil J, Mayard G, Louis-Charles J, Addiss D (1998) Filarial elephantiasis among Haitian women: social context and behavioural factors in treatment. Trop Med Int Health 3: 467–473. 9657509 10.1046/j.1365-3156.1998.00238.x

[2] Person B, Addiss D, Bartholomew LK, Meijer C, Pou V, et al (2008) “Can it be that God does not remember me”: a qualitative study on the psychological distress, suffering, and coping of Dominican women with chronic filarial lymphedema and elephantiasis of the leg. Health Care Women Int 29: 349–365. 18389432 10.1080/07399330701876406

[3] WHO (2010) Global Programme to Eliminate Lympathic Filariasis. Wkly Epidemiol Rec 85: 365–372. 20853547

[4] Galvez Tan JZ (2003) The elimination of lymphatic filariasis: A strategy for poverty alleviation and sustainable development - Perspectives from the Philippines. Filaria J 2: 12. 12914666 10.1186/1475-2883-2-12 PMC183860

[5] WHO (2007) Global Programme to Eliminate Lympathic Filariasis. Wkly Epidemiol Rec 82: 361–380. 17948605

[6] Raccurt C, Hodges W, Boncy J (1983) [Lymphatic filariasis in Haiti. Results of treatment with diethylcarbamazine in the town of Limbe]. Bull Soc Pathol Exot Filiales 76: 172–177. 6347417

[7] Raccurt CP (1999) [Filariasis in Haiti: a century of history]. Bull Soc Pathol Exot 92: 355–359. 10690476

[8] Beau de Rochars MV, Milord MD, St Jean Y, Desormeaux AM, Dorvil JJ, et al (2004) Geographic distribution of lymphatic filariasis in Haiti. Am J Trop Med Hyg 71: 598–601. 15569791

[9] WHO (2000) Preparing and implementing a national plan to eliminate lymphatic filariasis. WHO/CDS/CPE/CEE/2000.15. Available: http://whqlibdoc.who.int/hq/2000/who_cds_cpe_cee_2000.15.pdf. Accessed 17 June 2014.

[10] Drexler N, Washington CH, Lovegrove M, Grady C, Milord MD, et al (2012) Secondary mapping of lymphatic filariasis in Haiti-definition of transmission foci in low-prevalence settings. PLoS Negl Trop Dis 6: e1807 10.1371/journal.pntd.0001807 23071849 PMC3469481

[11] Beau de Rochars M, Kanjilal S, Direny AD, Radday J, Lafontant JG, et al (2005) The Léogâne, Haiti demonstration project: decreased microfilaremia and program costs after three years of mass drug administration. Am J Trop Med Hyg 73: 888–894. 16282299

[12] Grady CA, Beau de Rochars M, Direny AN, Orelus JN, Wendt J, et al (2007) Endpoints for lymphatic filariasis programs. Emerg Inf Dis 13: 608–610. 10.3201/eid1304.061063 PMC2725965 17553278

[13] Mathieu E, Lammie PJ, Radday J, Beach MJ, Streit T, et al (2004) Factors associated with participation in a campaign of mass treatment against lymphatic filariasis, in Léogâne, Haiti. Ann Trop Med Parasitol 98: 703–714. 15509424 10.1179/000349804X3135

[14] McLaughlin SI, Radday J, Michel MC, Addiss DG, Beach MJ, et al (2003) Frequency, severity, and costs of adverse reactions following mass treatment for lymphatic filariasis using diethylcarbamazine and albendazole in Léogâne, Haiti, 2000. Am J Trop Med Hyg 68: 568–573. 12812348 10.4269/ajtmh.2003.68.568

[15] Mathieu E, Deming M, Lammie PJ, McLaughlin SI, Beach MJ, et al (2003) Comparison of methods for estimating drug coverage for filariasis elimination, Leogane Commune, Haiti. Trans Roy Soc Trop Med Hyg 97: 501–505. 15307410 10.1016/s0035-9203(03)80006-8

[16] Worrell C, Mathieu E (2012) Drug coverage surveys for neglected tropical diseases: 10 years of field experience. Am J Trop Med Hyg 87: 216–222. 22855750 10.4269/ajtmh.2012.12-0167 PMC3414555

[17] Won KY, Beau de Rochars M, Kyelem D, Streit TG, Lammie PJ (2009) Assessing the impact of a missed MDA in Haiti. PLoS Negl Trop Dis 3: e443 10.1371/journal.pntd.0000443 19707279 PMC2724684

[18] Champetier de Ribes G, Fline M, Desormeaux AM, Eyma E, Champagne C, et al (2005) [Intestinal helminthiasis in school children in Haiti in 2002]. Bull Soc Pathol Exot 98: 127–132. 16050381

[19] Beau de Rochars M, Direny AD, Roberts JM, Addiss DG, Radday J, et al (2004) Community-wide reduction in prevalence and intensity of intestinal helminths as a collateral benefit of lymphatic filariasis elimination programs. Am J Trop Med Hyg 71: 466–470. 15516644

[20] Goldman AS, Brady MA, Direny A, Desir L, Oscar R, et al (2011) Costs of integrated mass drug administration for neglected tropical diseases in Haiti. Am J Trop Med Hyg 85: 826–833. 22049035 10.4269/ajtmh.2011.10-0635 PMC3205627

[21] Centers for Disease Control and Prevention (2010) Update: cholera outbreak—Haiti, 2010. MMWR 59: 1473–1479.21085088

[22] Centers for Disease Control and Prevention (2013) Mass Drug Administration for the Elimination of Lymphatic Filariasis — Port-au-Prince, Haiti, 2011–2012. MMWR 62: 466–468. 23760187 PMC4604845

[23] Talbot JT, Viall A, Direny A, de Rochars MB, Addiss D, et al (2008) Predictors of compliance in mass drug administration for the treatment and prevention of lymphatic filariasis in Léogâne, Haiti. Am J Trop Med Hyg 78: 283–288. 18256430

[24] Boyd A, Won KY, McClintock SK, Donovan CV, Laney SJ, et al (2010) Factors associated with continuing transmission of lymphatic filariasis in Léogâne, Haiti. PLoS Negl Trop Dis 4: e640 10.1371/journal.pntd.0000640 20351776 PMC2843627

[25] Cantey PT, Rout J, Rao G, Williamson J, Fox LM (2010) Increasing compliance with mass drug administration programs for lymphatic filariasis in India through education and lymphedema management programs. PLoS Negl Trop Dis 4: e728 10.1371/journal.pntd.000072 20628595 PMC2900179

[26] Dembele B, Coulibaly YI, Dolo H, Konate S, Coulibaly SY, et al (2010) Use of high-dose, twice-yearly albendazole and ivermectin to suppress Wuchereria bancrofti microfilarial levels. Clin Infect Dis 51: 1229–1235. 21039220 10.1086/657063 PMC3106228

[27] Houston R (2000) Salt fortified with diethylcarbamazine (DEC) as an effective intervention for lymphatic filariasis, with lessons learned from salt iodization programmes. Parasitology 121 (Suppl) S161–S173. 11386687 10.1017/s0031182000007150

[28] Lammie P, Milner T, Houston R (2007) Unfulfilled potential: using diethylcarbamazine-fortified salt to eliminate lymphatic filariasis. Bull World Health Organ 85: 545–549. 17768503 10.2471/BLT.06.034108 PMC2636360

[29] Freeman AR, Lammie PJ, Houston R, Jooste PL, Lapointe MD, et al (2001) A community-based trial for the control of lymphatic filariasis and iodine deficiency using salt fortified with diethylcarbamazine and iodine. Am J Trop Med Hyg 65: 865–871. 11791989 10.4269/ajtmh.2001.65.865

[30] WHO (2008) Meeting of the International Taskforce for Disease Eradication - 11 October 2007. Wkly Epidemiol Rec 83: 77–88. 18309578

[31] Addiss DG, Louis-Charles J, Roberts J, LeConte F, Wendt JM, et al (2010) Feasibility and effectiveness of basic lymphedema management in Leogane, Haiti, an area endemic for Bancroftian filariasis. PLoS Negl Trop Dis 4: e668 10.1371/journal.pntd.0000668 20422031 PMC2857874

[32] Norões J, Dreyer G (2010) A mechanism for chronic filarial hydrocele with implications for its surgical repair. PLoS Negl Trop Dis 4: e695 10.1371/journal.pntd.0000695 20532225 PMC2879368

